# Mental Health Inequities among Transgender People in Aotearoa New Zealand: Findings from the Counting Ourselves Survey

**DOI:** 10.3390/ijerph17082862

**Published:** 2020-04-21

**Authors:** Kyle K.H. Tan, Sonja J. Ellis, Johanna M. Schmidt, Jack L. Byrne, Jaimie F. Veale

**Affiliations:** 1 School of Psychology, University of Waikato, Hamilton 3240, New Zealand; jackbyrne@trans-action.nz (J.L.B.); jveale@waikato.ac.nz (J.F.V.); 2 School of Education, University of Waikato, Hamilton 3240, New Zealand; sonjaell@waikato.ac.nz; 3 School of Social Sciences, University of Waikato, Hamilton 3240, New Zealand; jschmidt@waikato.ac.nz; 4 TransAction, Auckland 1025, New Zealand

**Keywords:** transgender, mental health inequity, psychological distress, depression, anxiety, age

## Abstract

There has been little international research looking at differences in mental health across different age groups. This study examines mental health inequities between transgender people and the Aotearoa/New Zealand general population from youth to older adulthood. The 2018 Counting Ourselves survey (*N* = 1178) assessed participants’ mental health using the Kessler Psychological Distress Scale (K10) and diagnoses of depression and anxiety disorders, questions that were the same as those used in the New Zealand Health Survey. Our results showed significant mean score differences for transgender people on K10, and these differences were almost two standard deviations higher than the general population (Cohen’s *d* = 1.87). The effect size differences, however, decreased from youth to older adults. Regression analyses indicated trans women were less likely to report psychological distress than trans men and non-binary participants. There was an interaction effect for age and gender, with lower psychological distress scores found for younger trans women but higher scores for older trans women. The stark mental health inequities faced by transgender people, especially youth, demonstrate an urgent need to improve the mental health and wellbeing of this population by implementing inclusive institutional practices to protect them from gender minority stress.

## 1. Introduction

Transgender (or trans) people are those whose gender does not correspond with their sex assigned at birth. In this article, we use transgender as an umbrella term to encompass trans men (those who identify as men but were assigned female at birth), trans women (those who identify as women but were assigned male at birth), and non-binary people (those whose gender is neither man nor woman) [1]. These broad descriptions include identities formed in both Western and non-Western cultural contexts. The primary ethnic groups in Aotearoa/New Zealand are New Zealand European (also known as Pākehā and equating to the term “White” in many other English-speaking places), indigenous Māori, Pacific Island, and Asian. Māori terms that encompass gender diversity include whakawahine, takatāpui, and tangata ira tāne [2]. The significant Pacific population also means that New Zealanders are relatively familiar with terms such as the Samoan identity, fa’afafine [3].

Increasing international evidence has shown that transgender people experience significant mental health inequities when compared to the cisgender population (people whose gender aligns with their sex assigned at birth). This has been demonstrated across North America [4,5,6,7], South America [8], Europe [9], Oceania [10,11], and Asia [12]. A population-based health survey in the United States, the 2014–2016 Behavioral Risk Factor Surveillance System (BRFSS) survey, found transgender people had a higher self-rating of mental distress (20% vs. 11%) and were more likely to report a depression diagnosis (27% vs. 17%) than the cisgender population [4]. The Aotearoa/New Zealand population-based adolescent health survey, Youth’12, reported an almost fourfold increase in depressive symptoms (42% vs. 12%), a twofold increase in non-suicidal self-injury risk in the past year (46% vs. 23%), and a fivefold increase in suicide attempts in the past year (20% vs. 4%) among transgender high school students compared to their cisgender counterparts [10]. The Youth’12 study, however, focused only on adolescents and was limited to 96 transgender participants.

Public health literature commonly defines *health inequalities* as differences in health outcomes between groups or specific cultures within a population [13]. The term *health inequities* reflects a social justice lens and foregrounds the impacts of unjust social norms that prevent a population from attaining their full health potential [13,14]. In this instance, the systemic difference in health status between transgender and cisgender populations are affected by cisgenderism, a prejudicial norm that asserts that there are only two valid genders (i.e., man and woman) which correspond to one’s assigned sex at birth [15,16]. The Gender Minority Stress Theory posits that cisgenderism leads to a form of stress that is specific to transgender people, and that elevated mental health concerns among this population are due to their experiences of distal (e.g., external discrimination) and proximal (e.g., internalised transphobia) stressors [16,17,18].

Evidence from recent studies in the United States showed younger transgender participants reporting higher levels of mental health concerns, and that these people were more likely to experience gender minority stressors, such as discrimination and internalised stigma (a form of internalised discomfort with one’s transgender identity that is stimulated by distal stressors) than older participants [19,20]. Realising these generational differences in mental health, we extended existing transgender mental health studies that have only examined specific age groups, such as youth [1,6,9,10] and older adults [21] by assessing mental health inequities across the lifespan from adolescence to older adults.

Findings of mental health differences among gender groups within the transgender population (i.e., trans men, trans women, and non-binary people) have been mixed. While some studies documented significantly poorer mental health outcomes for non-binary participants relative to trans men and trans women [6,9], their results were restricted to transgender youth. Other studies have recruited transgender people of all age groups and adjusted for the effects of demographic variables, such as age, in identifying gender differences; contrary to the transgender youth studies, these found that non-binary participants had better mental health than trans men and trans women [5,22]. Given these discrepancies, this study explores the relationship of gender, along with age, on mental health, and does so in more detail than previous studies by also assessing the interaction effect between these variables.

## 2. Materials and Methods

### 2.1. Procedure

Counting Ourselves: the Aotearoa New Zealand Trans and Non-Binary Health Survey was a survey of transgender and non-binary people who were at least 14 years old and resided in Aotearoa/New Zealand. The recruitment strategy focused on ensuring that there was adequate representation of the diversity among transgender people. To do this, transgender people of older age groups, Māori, Pacific, and Asian ethnic groups, and those residing in rural areas were invited to share quotes about the importance of the survey to them, and have their images drawn alongside their quotes in posters. These posters were shared on the project website and distributed through social media. Participants were also recruited through billboards and word-of-mouth with help from our networks of transgender community organisations, academic researchers, and health professionals working in transgender healthcare (see [23] for more details). The study received ethical approval from the New Zealand Health and Disability Ethics Committee (18/NTB/66/AM01) and was open for participation from June to September 2018 in both online and paper forms.

### 2.2. Participants

The survey had 1380 initial responses. After filtering out responses that were duplicates (*n* = 22), not from Aotearoa/New Zealand (*n* = 12), less than the age 14 requirement (*n* = 2), did not complete the initial demographic section to confirm that they were transgender (*n* = 161), or were not genuine (*n* = 5) (see [23] for more details), the final sample for analysis included 1178 transgender people whose ages ranged from 14 to 83 years (M = 29.5, SD = 13.3). However, not all participants completed the whole survey due to attrition over a long survey; more than three quarters (*n* = 905) completed the mental health section of the survey.

**Figure 1:**
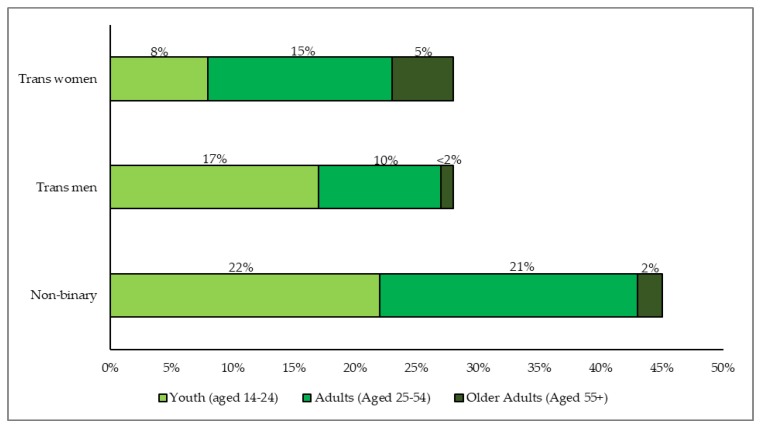
The distribution of gender groups across the lifespan among Counting Ourselves participants.

Most participants were New Zealand European/Pākehā (82%), followed by Māori (14%), Samoan (2%), Tongan (1%), Chinese (1%), and Filipino (1%). When compared to the estimates of the Aotearoa/New Zealand general population, the Counting Ourselves survey had relatively more New Zealand European/Pākehā and Māori participants and relatively fewer Asian participants. Our sample had many non-binary people (45%; with 76% of this group having been assigned female at birth), and similar proportions of trans women (28%) and trans men (27%) (Note that these demographic details may differ slightly from the published findings from the same survey dataset ([23]) which were weighted to by ethnic groups to match the Aotearoa/New Zealand population.). Figure 1 presents the distribution of gender groups across the lifespan, with three gender groups displaying distinctive age structures. There was a relatively higher proportion of trans women among older adults and higher proportions of trans men and non-binary people among younger participants.

### 2.3. Population Comparisons

Existing population-based health surveys in Aotearoa/New Zealand, such as the New Zealand Health Survey (NZHS) 2016/17 of 13,598 people aged 15 and above, did not collect data about whether someone was transgender [24]. The NZHS 2016/17 employed the probability proportional to size sampling and also applied weighting to ensure data were representative of the New Zealand demographic distribution [24].

### 2.4. Measures

#### 2.4.1. Gender

Participants were classified into three gender groups (trans men, trans women, and non-binary) using two items that asked about sex assigned at birth and current gender identities. We classified participants as trans men if they reported man, trans man, or transsexual as their gender and were assigned female at birth. Trans women were participants who selected woman, trans woman, or transsexual and were assigned male at birth. All other participants were classified as non-binary.

#### 2.4.2. Number of Years Living Full-Time in Affirmed Gender

Trans women and trans men were asked to list the age of started living full-time as a woman or man. The number of years lived full-time in their affirmed gender for these groups was calculated by subtracting the age they started living full-time as a woman or man from their current age.

#### 2.4.3. Mental Health Diagnoses

We used the same measures of mental health diagnoses found in questions from the NZHS 2016/17 [24]. Participants were asked whether they had ever been told by a doctor that they had depression or an anxiety disorder, with “yes” and “no” response options. We avoided using the term “disorder”, given the history of transgender people resisting having their gender diversity or health needs framed pathologically in this way. The term appears in the text where this study used questions from a population-based survey to compare the prevalence of depression and anxiety diagnoses among transgender people with the general population estimates.

#### 2.4.4. Psychological Distress

Psychological distress was measured in our study and the New Zealand Health Survey using the Kessler Psychological Distress scale (K10). This scale measures the presence of non-specific psychological distress symptoms in the past 4 weeks, using 10 items with a five-point response scale, from none of the time (0) to all of the time (4) [25]. Scores can range from 0 to 40, with higher scores indicating someone is manifesting higher levels of behavioural, emotional, cognitive, and/or psychophysiological symptoms of psychological distress. A score of 12 or more suggests the presence of high levels of psychological distress symptoms [24]. The K10 has demonstrated sound validity in screening for cases of mood and anxiety disorders among the Aotearoa/New Zealand general population [26]. In the current dataset, the internal consistency of the K10 was high (α = 0.94).

### 2.5. Data Analysis

All statistical analyses were performed in IBM SPSS Statistics version 25 (IBM, Armonk, NY, USA). The percentage of missing data for each K10 item ranged from 0.2% to 1.1%, and these missing values were imputed using the expectation maximisation method in which values were estimated by regression methods based on means and covariances of available data [27]. We identified mental health inequities between transgender participants and the general population by conducting independent sample t-tests to assess the differences in means of the psychological stress scores. Chi-square goodness of fit tests were used to compare the observed proportion for dichotomous mental health diagnoses with the expected value of the general population. Cohen’s d and risk ratio estimates were used to measure the effect size differences of mental health inequities.

The multivariate relationships among mental health, gender, and age were explored by employing the linear and logistic regression analyses with gender and age (and their interaction) predicting mental health diagnoses and K10 psychological distress. Low variance inflation factors (VIFs = 1.09) of age and gender variables in our sample indicated that the assumption of independence was not violated. An alpha level of *p* < 0.05 was used to determine statistical significance for all analyses in this study.

## 3. Results

Nearly three quarters (72%) of participants manifested high or very high psychological distress symptoms (i.e., a score of 12 or more on the K10 scale). Nearly two-thirds reported having been told by a doctor that they had depression (65%), and over half (56%) had been told by a doctor they had an anxiety disorder.

### 3.1. Mental Health Inequities

New Zealand general population-based estimates were for those aged 15 and older, so we excluded data from 14-year-old participants (*n* = 25) in this analysis. Table 1 outlines comparisons between Counting Ourselves and the New Zealand Health Survey for K10 psychological distress scores and mental health diagnoses. The inequity in psychological distress scores was particularly prominent, with effect size differences of almost two standard deviations for the overall sample. Considerable differences were also found for rates of being diagnosed with a mental health disorder, with participants having almost three times the risk of reporting a lifetime depression diagnosis and a more than five times greater risk of reporting an anxiety disorder diagnosis.

### 3.2. Age Group Differences

**Figure 2:**
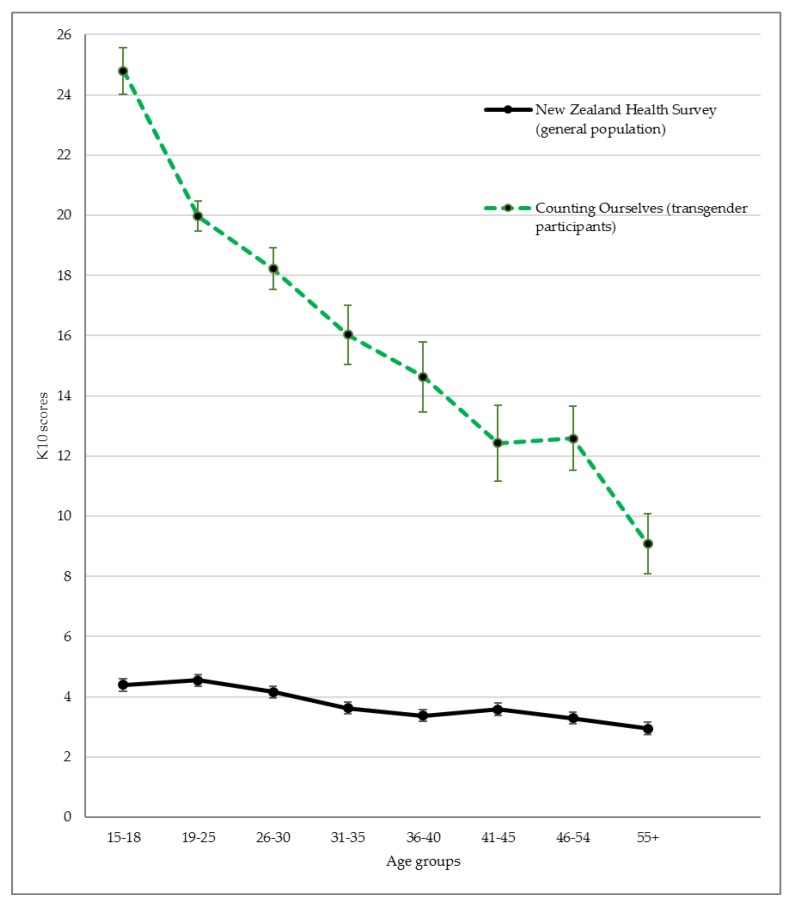
The inequities in K10 psychological distress scores between Counting Ourselves participants and the Aotearoa/New Zealand general population across age groups.

Figure 2 illustrates the extent of inequities for psychological distress scores across age groups. This shows a reduction of effect size differences from younger to older age groups.

A similar trend was observed for the mental health diagnoses findings in Table 1. For depression, risk ratios varied from more than seven for 15- to 18-year-olds, to almost three times for those aged 55 and above. Transgender youth aged 15 to 18 years had ten times the risk of reporting having a diagnosis of anxiety disorder.

### 3.3. Gender and Age Differences

Regression analyses revealed strong negative associations of age with an anxiety diagnosis and psychological distress scores (see Table 2; This analysis included 14-year-old participants.). There were statistically significant main effects for gender for depression and anxiety diagnoses in the age-adjusted models, but not for psychological distress. Table 2 reports the differences in odds ratios and predicted scores from the respective gender groups to the reference category (trans women). The regression model noted trans men and non-binary participants had significantly higher odds of depression and anxiety diagnoses than trans women, independently of these groups. There were, however, no significant gender differences, and the interaction effect of age and gender found for mental health diagnoses in the age-interaction models. The results of these interaction effects are presented in Table S1.

**Figure 3:**
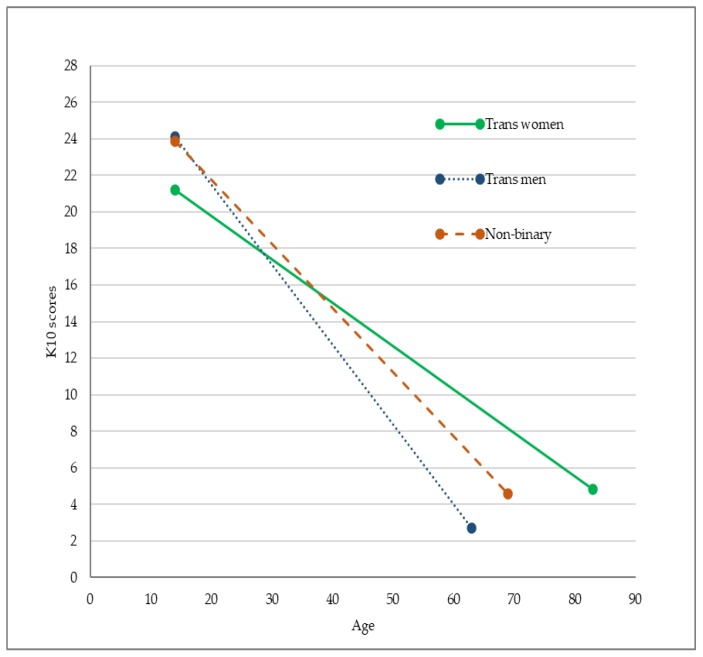
The interaction effect of age and gender on K10 psychological distress scores.

The interaction effect between age and gender, however, was a statistically significant predictor of psychological distress. Compared to trans women, our findings in the age interaction model showed trans men and non-binary participants to have 5.67 and 4.26 points higher average psychological distress scores, respectively. We illustrated the interaction effect of gender and age on psychological stress scores in Figure 3. The presence of more steeply negative regression lines for trans men and non-binary participants indicated a more rapid decrease in psychological distress scores from younger to older ages within these gender groups when compared to trans women.

Upon recommendations from anonymous reviewers, we conducted supplementary analyses, including the effect of the number of years living in the affirmed gender, to examine whether there were any changes to the effects of age and gender on transgender people’s mental health (see Table S2). As we did not ask our non-binary participants the age first lived in their affirmed gender, this gender group was excluded from this analysis. We found that statistically significant gender differences for depression and anxiety diagnoses remained on the supplementary analysis, as well as for the interaction effect of age and gender on psychological distress scores.

## 4. Discussion

### 4.1. Population-Based Comparisons

Using data from the Counting Ourselves survey, this study explored the extent of mental health inequities that transgender people in Aotearoa/New Zealand face. There was a ninefold increase in the manifestation of high or very high psychological distress symptoms when comparing transgender participants (72%) to the general population (8%). While some studies have found high levels of psychological distress among transgender participants [19,28], the other studies we could find using the Kessler Psychological Distress Scale were the United States Trans Survey 2015 using the shortened version—K6 [7], and an Australian study [29]. Comparatively, our sample had a higher prevalence of serious psychological distress than that reported in the United States study (44% vs. 39%; measured with K6) (We used same items that are in the K6 scale to compare with the findings of the 2015 U.S. Trans Survey. A serious psychological distress level was identified by a total score of 13 or more on the K6), and high or very high levels of psychological distress compared to the Australian study (72% vs. 46%; measured with K10) community-based studies.

Our transgender participants were also more likely to report having received a mental health diagnosis by a health professional than the general population, with approximately fourfold differences for depression and more than fivefold differences for anxiety disorders. The prevalence of depression (66%) and anxiety disorders (55%) among our Aotearoa/New Zealand transgender participants were also higher than those reported in the United States (47% for depression and 42% for anxiety disorders) [22] and Australian studies (57% for depression and 40% for anxiety disorders) [11]. The discrepancy in prevalence could be due to the older average age of transgender participants in the other studies; these warrant further investigation. The mental health inequities between transgender and cisgender participants found in this study are consistent with and add to the body of evidence confirming the deleterious impacts of gender minority stress [16,17,18]. Our questions on depression and anxiety asked about the lifetime prevalences of these diagnoses; we are aware that these might be prone to recall bias, and that we cannot necessarily infer one’s current mental health status from these particular questions in the way that we can from our psychological distress questions. Nevertheless, a strength of our study is that it highlights inequities with the general population for both current mental health status (psychological distress in the past 4 weeks) and lifetime meantal health status (depression and anxiety diagnoses).

### 4.2. Age Comparisons

Our study also looked at inequities (i.e., comparisons with the general population) for transgender people across different age groups from youth to older adults. Other studies have found that younger transgender participants reported a higher prevalence of mental health diagnoses [11] and psychological distress symptoms [28,29], but we also know that in the general population, youth were at higher risk of mental health difficulties than adults and older adults ([30]; see also Figure 2). The 2015 United States Transgender Survey also compared the prevalence of serious psychological distress across age groups relative to the general population [7]. Similar to our findings, James et al. reported higher inequities for transgender participants aged 18 to 25 (53% vs 10%, RR = 5.3) than those aged 65 and older (8% vs 2%, RR = 4.0) [7]. We are not aware of any studies that have examined inequities between transgender people and the general population in the prevalence of depression and anxiety diagnoses for younger transgender youth; other studies on this topic only recruited participants of 18 years or older [4,7,11,22], although other studies have found that adolescent trans youth were more likely to report self-depression and anxiety symptoms than older transgender youth (e.g., [31]). It is important to note, however, that while mental health inequities faced by older transgender people were less, they still faced substantial mental health inequities compared to the older aged general population.

Examining research on gender minority stressors may help to explain these mental health inequities across different age groups. A United States online survey revealed younger transgender people were more vulnerable to the negative mental health effects of gender minority stressors than their older counterparts [28]. Jackman et al. in the United States found lower levels of internalised stigma among older transgender people, and suggested that this may be due to them having developed better coping skills and social support systems (maturation effect) in counteracting the effects of gender minority stressors [19]. Longitudinal research is needed to uncover the ways that transgender people build resilience and support over time that may provide mental health benefits.

While our study has identified age as an important demographic factor in predicting transgender people’s mental health, we could not be certain whether the mental health differences across age groups represented changes as this population grew older (aging effect), their development of the ability to cope with gender minority stressors later in life (maturation effect), or whether they were the result of historical and social contexts that occurred for specific age groups (cohort effects). A comprehensive understanding of the mental health status of transgender people of different age groups would require an examination on how minority stress and resilience for transgender people changes over the life span [14,19,32].

### 4.3. Gender Group Comparisons

In our sample, trans women were over-represented in older age groups (aged 55 and above), while the younger participants were more likely to be trans men or non-binary. A population-based study in United States [4] and community-based studies in United Kingdom [1,5], United States [22], and Canada [6] demonstrated similar findings, with trans men and non-binary individuals being more common in the trans youth samples of these studies. Because of these differences, we included age as a variable in the regression models that examined gender group differences.

After controlling for the age effect, we found that trans men reported higher prevalences of depression and anxiety diagnoses. This aligns with the findings of other population-based studies [4] and community-based studies that employed convenience sampling [1,28]. Such findings might be explained partly by research on differential experiences of gender minority stressors among gender groups within the transgender population which have found trans men to be more likely to report sexual abuse and domestic violence [1], and discrimination when accessing employment and healthcare services [28]. One study in the United Kingdom noted trans men and women were no more or less likely to seek professional help for mental health problems [1].

In our sample, non-binary participants were more likely than trans women to have been diagnosed with depression and anxiety disorders. Findings from previous studies of non-binary people’s mental health compared to the other two gender groups have been mixed. Studies of transgender youth [6,9] and a population-based study of adults in the United States [4] found that non-binary participants reported higher levels of mental health concerns. Crissman et al. also found that this difference held after accounting for age differences among the gender groups. This finding, however, was not replicated in a recent United States community-based study which found that non-binary participants had lower odds of reporting depression and anxiety diagnoses by health professionals compared to trans men and trans women, even after adjusting for the effects of demographic variables, such as age [22]. This discrepancy could be due to Reisner and Hughto’s study having an equal proportion of non-binary participants who were assigned male and female at birth, respectively [22], whereas our study and the other studies had a higher proportion of non-binary people assigned female at birth.

To extend our knowledge about this topic, we were the first study to also examine the interaction effect between age and gender on transgender people’s mental health outcomes. It is important to examine the interaction of independent variables in regression analyses, because omitting the interaction effect can lead to a biased estimation of model parameters when an interaction effect is present [33]. Notably, gender differences in mental health diagnoses were no longer statistically significant when we included the age and gender interaction term in the models. This finding suggests that when the interaction effect of age and gender is estimated, trans men and non-binary participants no longer had significantly higher rates of having been diagnosed with depression and anxiety disorders relative to trans women.

Our exploratory finding of an interaction effect on the K10 scale, however, suggested that the relationship between age and psychological distress scores varied across different gender groups. For instance, we found that younger trans women reported less psychological distress compared to trans men and non-binary people of the same age groups. This trend changed for older age groups, where trans women reported more symptoms of psychological distress than other gender groups. This interaction effect remained statistically significant after adjusting for the number of years lived in the affirmed gender (see Table S2), suggesting that the length of time living in the affirmed gender is not the reason for this difference. Increased rejection and less social support for older trans women may explain this finding. A United States study that examined mental health of trans women across the life span found older trans women were less likely to have stable relationships with family members and friends [32]. More research is needed to replicate this interaction finding and further explore the reasons for it, but this research suggests a clear need to consider interaction effects when exploring how age and gender are related to mental health for transgender people.

There are other within-group differences that can be assessed of our data that is beyond the scope of this paper, including race/ethnicity, disability status, and cultural connectedness, and future research should consider the variations of mental health outcomes for these different subgroups of transgender people.

## 5. Limitations

It is difficult to determine the degree to which the convenience sample used in Counting Ourselves was representative of transgender people in Aotearoa/New Zealand. While the use of a recruitment strategy to promote the survey via online platforms and community organisations has allowed us to achieve a large sample that enabled us to conduct comparisons based on age and gender groups, it risks under-representing transgender people who were not active in transgender communities, including those who transitioned a long time ago. This may have contributed to the relatively small sample of older (age 55 and older) transgender participants in our study. However, clinically based research in Aotearoa/New Zealand also found a relatively lower proportion of older transgender people accessing gender-affirming care [34], and this demographic profile has also been seen in a recent population-based study in the United States [4]. Relative to the overall population in Aotearoa/New Zealand, Counting Ourselves achieved a sample size that was many times larger than other recent national transgender surveys that employed convenience sampling [6,7,11].

## 6. Conclusions

Counting Ourselves is the first large quantitative study in Aotearoa/New Zealand to describe mental health inequities for transgender people from youth to older adulthood. Our findings indicate significant mental health inequities faced by transgender participants, which is consistent with the Gender Minority Stress Theory. This study is the first, to our knowledge, to explore the interaction effect of age and gender on mental health outcomes, finding that trans men and non-binary participants had larger changes in psychological distress from youth to older adulthood.

## 7. Study Implications 

To ameliorate the risk of mental health problems among transgender people, we recommend immediate actions on behalf of the policy makers in Aotearoa/New Zealand to identify transgender people as a named priority in mental health policies, for mental health professionals to receive training on cultural competency for working with transgender communities, and for funding for peer support and other wellbeing initiatives led by transgender communities [35]. Mental healthcare providers and service workers should be aware of the very high risk of psychological distress and mental health problems faced by transgender people. Where there are gaps in delivering care that is appropriate to transgender people, relevant training should be required so that mental healthcare workers can better serve the needs of this population. Mental health inequities affecting transgender people need to be understood in relation to gender minority stress as a social determinant, as studies have shown that discrimination, stigma, and social exclusion against transgender people could limit their abilities to access services that are crucial to health and wellbeing, including education, employment, and healthcare services [36,37]. In line with the international human rights standards [38], institutions across different settings, such as schools, employers, and government agencies have an obligation to generate a safe and welcoming environment for transgender people, as well as to implement trans-inclusive policies and interventions to protect this population from exposure to gender minority stressors (e.g., discrimination and workplace bullying) [23].

While these inequities in mental health outcomes apply to transgender people of all genders and ages, it is important to recognise the specific gender minority stress experiences and mental health needs of transgender youth who have been found to be at extreme risk of developing mental health problems. With the recent surge of negative media aimed at negating transgender people’s lived experiences and discrediting the merits of gender-affirming care, there is still much work to be done to ensure this population can enjoy human rights to health without being jeopardised by discrimination or stigma. Healthcare providers should advocate for equitable access to mental healthcare services and gender-affirming care for transgender people to attain the highest standard of health [38,39]. It is also important for health professionals working with transgender youth to respect their privacy, self-determination, autonomy, as well as their evolving capacity to make informed decisions about their health [40].

Transgender people remain understudied in Aotearoa/New Zealand, and we urge government agencies to include standardised survey items to identify transgender people in population-based surveys. Health surveillance efforts that are done in collaboration with transgender communities will allow health professionals, policy makers, and transgender people themselves to monitor and evaluate efforts to achieve health equity. More specific recommendations to improve transgender people’s mental health and wellbeing in Aotearoa/New Zealand can be read from the published report [23]. Finally, our findings of differential levels of psychological distress across various gender and age groups also show that future research should consider the effect of age when examining gender group differences in mental health among transgender people.

## Figures and Tables

**Table 1:** Mental health characteristics across age groups and comparisons with New Zealand Health Survey 2016/7 (age 15+).

Age Groups	Counting Ourselves		NZHS 2016/7		
	*n*	%/M (SD)	%/M (SD)	t-Test/Chi-Square Statistics	Effect Size
Depression (Yes; No)	854	65.7%	16.7%	49.45 **	RR = 2.88 (2.47, 6.12)
15−18	112	53.6%	6.8%	52.11 **	RR = 7.71 (3.69, 16.12)
19−25	288	71.5%	14.7%	64.85 **	RR = 4.80 (2.96, 7.78)
26−30	149	72.5%	18.2%	59.75 **	RR = 4.06 (2.63, 6.27)
31−35	75	69.3%	15.4%	59.85 **	RR = 4.60 (2.83, 7.37)
36−40	51	70.6%	17.5%	57.59 **	RR = 3.94 (2.55, 6.10)
41−45	43	62.8%	19.3%	40.02 **	RR = 3.32 (2.15, 5.11)
46−54	64	56.3%	18.9%	29.21 **	RR = 2.95 (1.90, 4.58)
55+	72	50.0%	17.5%	23.24 **	RR = 2.78 (1.75, 4.41)
Anxiety (Yes; No)	853	55.2%	10.3%	44.49 **	RR = 5.50 (2.98, 10.16)
15−18	117	53.8%	5.4%	57.72 **	RR = 10.80 (4.51, 25.86)
19−25	291	66.0%	11.7%	61.29 **	RR = 5.50 (3.18, 9.52)
26−30	149	58.4%	12.6%	44.22 **	RR = 4.46 (2.62, 7.61)
31−35	74	59.5%	9.1%	56.63 **	RR = 6.67 (3.50, 12.69)
36−40	47	55.3%	10.7%	43.78 **	RR = 5.00 (2.79, 8.98)
41−45	43	32.6%	11.8%	12.65 **	RR = 2.75 (1.51, 5.01)
46−54	60	40.0%	11.3%	22.13 **	RR = 3.64 (1.98, 6.67)
55+	72	29.2%	9.4%	13.00 **	RR = 3.22 (1.61, 6.45)
K10 (0−40)	886	17.86 (9.56)	3.54 (5.12)	83.11 **	*d* = 1.87 (1.80, 1.93)
15−18	124	24.80 (8.65)	4.39 (5.31)	42.78 **	*d* = 2.84 (2.67, 3.02)
19−25	298	19.96 (8.49)	4.55 (5.96)	44.61 **	*d* = 2.10 (1.99, 2.22)
26−30	152	18.22 (8.60)	4.16 (5.93)	29.22 **	*d* = 1.90 (1.75, 2.06)
31−35	75	16.03 (8.89)	3.62 (5.05)	21.26 **	*d* = 1.72 (1.49, 1.94)
36−40	53	14.63 (8.24)	3.38 (4.93)	16.60 **	*d* = 1.66 (1.39, 1.93)
41−45	46	12.43 (8.17)	3.58 (5.24)	11.46 **	*d* = 1.29 (1.00, 1.58)
46−54	64	12.58 (9.49)	3.29 (4.88)	15.22 **	*d* = 1.23 (0.98, 1.48)
55+	74	9.08 (7.57)	2.95 (4.49)	11.73 **	*d* = 0.98 (0.76, 1.21)

Significant difference ** *p* < 0.01.

**Table 2:** Regression analyses of gender and age on mental health variables among Counting Ourselves age 14+ participants.

Variables	Depression Diagnosis		Anxiety Diagnosis		K10 Psychological Distress			
	Age-adj Model		Age-adj Model		Age-adj Model		Age-int Model	
	Wald Statistics	OR (95% CI)	Wald Statistics	OR (95% CI)	Wald Statistics	b (95% CI)	Wald Statistics	b (95% CI)
*Age*	0.82	1.00 (0.98, 1.01)	15.18 **	0.98 (0.97, 0.99)	189.20 **	−0.31 (−0.35, −0.27)	195.66 **	−0.24 (−0.30, −0.18)
*Gender*	7.76 *		21.121 **		0.87		9.59 **	
Trans women		1.00 (ref)		1.00 (ref)		1.00 (ref)		1.00 (ref)
Trans men	7.38 **	1.72 (1.16, 2.55)	17.91**	2.29 (1.56, 3.36)	0.13	−0.28 (−1.85, 1.29)	8.39 **	5.67 (1.83, 9.51)
Non-binary	4.00 *	1.42 (1.01, 2.01)	15.03**	1.97 (1.40, 2.77)	0.24	0.36 (−1.06, 1.77)	5.80 *	4.27 (0.79, 7.74)
*Gender x Age*	-	-	-	-	-	-	11.90 **	
Trans women	-	-	-	-	-	-		1.00 (ref)
Trans men	-	-	-	-	-	-	10.27 **	−0.20 (−0.32, −0.08)
Non-binary	-	-	-	-	-	-	5.06 *	−0.11 (−0.21, −0.02)
*n*	870		871		904			

Trans women were used as a reference group for comparison. Age-adj model = Regression models adjusted for the effects of age. Age-int model = Regression models examining the interaction effect of gender groups and age. Significant difference * *p* < 0.05 ** *p* < 0.01.

## Supplementary Materials

The following are available online at https://www.mdpi.com/1660-4601/17/8/2862/s1, Table S1: Regression analyses of gender and age on mental health variables among Counting Ourselves age 14+ participants. Table S2. Regression analyses of number of years living in affirmed gender, gender, and age on mental health variables among Counting Ourselves age 14+ trans men and trans women participants.
