# Supplementary material for: Mental Health Inequities among Transgender People in Aotearoa New Zealand: Findings from the Counting Ourselves Survey
Source: Int J Environ Res Public Health. 2020 Apr 21;17(8):2862. doi: 10.3390/ijerph17082862 (PMC7216046; doi:10.3390/ijerph17082862)
Supplement: Supplementary file 1 [file ijerph-17-02862-s001.pdf]

Table S1. Regression analyses of gender and age on mental health variables among Counting Ourselves age 14+ participants.

	Depression diagnosis		Anxiety diagnosis	
	**Age-int Model**		**Age-int Model**	
**Variables**	**Wald statistics**	**OR (95% CI)**	**Wald statistics**	**OR (95% CI)**
**Age**	3.57	0.99 (0.97, 1.00)	9.97 **	0.97 (0.96, 0.99)
**Gender**	0.42		1.26	
Trans women		1.00 (ref)		1.00 (ref)
Trans men	0.37	0.73 (0.27, 1.99)	0.89	1.59 (0.61, 4.16)
Non-binary	0.22	0.81 (0.34, 1.94)	1.01	1.56 (0.66, 3.73)
**Gender x Age**	3.69		0.69	
Trans women		1.00 (ref)		1.00 (ref)
Trans men	2.96	1.03 (1.00, 1.06)	0.62	1.01 (0.98, 1.04)
Non-binary	1.67	1.02 (0.99, 1.04)	0.28	1.01 (0.98, 1.03)

Trans women were used as a reference group for comparison. Age-int model = Regression models examining the interaction effect of age and gender groups.

Significant difference * $p < 0.05$ ** $p < 0.01$

Table S2. Regression analyses of number of years living in affirmed gender, gender, and age on mental health variables among Counting Ourselves age 14+ trans men and trans women participants.

	**Depression**		**Anxiety**		**K10**			
	**Age-adj Model**		**Age-adj Model**		**Age-adj Model**		**Age-int Model**	
**Variables**	**Wald statistics**	**OR (95% CI)**	**Wald Statistics**	**OR (95% CI)**	**Wald Statistics**	**B (95% CI)**	**Wald Statistics**	**B (95% CI)**
**Age**	0.23	1.00 (0.98, 1.01)	5.66 *	0.98 (0.96, 1.00)	31.44 **	-0.22 (-0.30, -0.15)	16.45 **	-0.18 (-0.27, -0.09)
**Years living in affirmed gender**	0.55	0.99 (0.96, 1.02)	1.20	0.98 (0.94, 1.02)	1.97	-0.10 (-0.25, 0.04)	1.63	-0.09 (-0.24, 0.05)
**Gender** [a]	8.48 **	2.09 (1.27, 3.43)	13.31 **	2.38 (1.50, 3.80)	0.01	-0.09 (-2.12, 1.93)	3.63	4.75 (-0.14, 9.63)
**Gender x Age** [a]	-	-	-	-	-	-	4.55 *	-0.16 (-0.31, -0.01)

Number of years since living in affirmed gender was calculated with the equation "current age - age of first started living as a woman or man". We did not ask non-binary participants their age of first living in affirmed gender so we could not calculate the number of years of living in affirmed gender for this gender group. [a] Trans women were used as a reference group for comparison. Age-adj model = Regression models adjusted for the effects of age. Age-int model = Regression models examining the interaction effect of age and gender groups.

Significant difference * $p < 0.05$ ** $p < 0.01$
